# [Xe(OTeF_5_)(py^F^)]^+^: a strong oxidizing xenonium(ii) teflate cation with N-donor bases†

**DOI:** 10.1039/d3cc05560d

**Published:** 2023-12-13

**Authors:** Ahmet N. Toraman, Lukas Fischer, Alberto Pérez-Bitrián, Anja Wiesner, Kurt F. Hoffmann, Sebastian Riedel

**Affiliations:** a Institut für Chemie und Biochemie, Freie Universität Berlin, Fabeckstraße 34/36 Berlin 14195 Germany s.riedel@fu-berlin.de; b Institut für Chemie, Humboldt-Universität zu Berlin Brook-Taylor-Straße 2 Berlin 12489 Germany alberto.perez-bitrian@hu-berlin.de

## Abstract

Herein we report on the formation of the adduct salts [Xe(OTeF_5_)(py^F^)][Al(OTeF_5_)_4_] (py^F^ = C_5_F_5_N, C_5_H_3_F_2_N) by abstraction of an –OTeF_5_ group from Xe(OTeF_5_)_2_ with the Lewis superacid Al(OTeF_5_)_3_ and subsequent adduct formation of the generated [XeOTeF_5_]^+^ cation with fluorinated pyridines. These salts represent the first xenonium cations with the weakly coordinating [Al(OTeF_5_)_4_]^−^ anion. The strong oxidizing property of these compounds is further assessed.

## Abstract 2

The strong oxidizing [Xe(OTeF_5_)(py^F^)]^+^ are the first xenonium cations stabilized by the [Al(OTeF_5_)_4_]^−^. They are formed by abstraction of a –OTeF_5_ group from Xe(OTeF_5_)_2_ by the Lewis superacid Al(OTeF_5_)_3_, and adduct formation with nitrogen bases.

## Graphical abstract

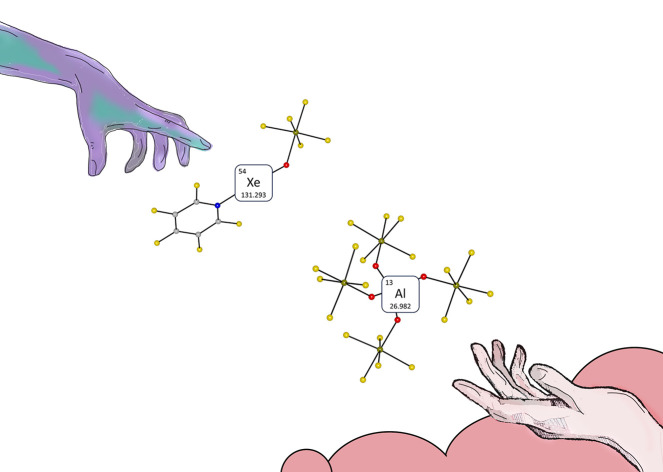

The strong oxidizing [XeF]^+^ cation exhibits a high Lewis acidity and therefore readily forms adducts with *e.g.* nitrogen bases that are resistant to oxidation.^1,2^ In fact, the chemistry of Xe–N species has mostly been realized by coordination of nitrogen-containing bases to stabilize the [XeF]^+^ cation, although they can be produced by the reaction of XeF_2_ with a suitable protic acid and the release of HF.^3^

The pentafluoroorthotellurate (teflate, –OTeF_5_) group resembles the fluoride ligand in its high electron withdrawing properties, but with a higher steric demand.^4,5^ Therefore, a variety of reactive species, including noble-gas compounds,^6^*e.g.* Xe(OTeF_5_)_2_ and Kr(OTeF_5_)_2_, have been synthesized.^7,8^ The teflate analogue of the [XeF]^+^ cation, namely [XeOTeF_5_]^+^, was first reported by Sladky in the reaction of FXeOTeF_5_ in combination with the Lewis acid AsF_5_, which forms the [XeOTeF_5_][AsF_6_] salt.^9^ Later it was shown, that this salt has a significant cation–anion interaction through a fluoride bridge.^10^ This interaction between the xenon(ii) centre and the [AsF_6_]^−^ anion is indicative of the high Lewis acidity of the [XeOTeF_5_]^+^ cation, similar to its well-known fluoride analogue [XeF]^+^. Efforts to isolate the free [XeOTeF_5_]^+^ cation in the solid state by utilizing weakly coordinating anions (WCAs) such as [Sb(OTeF_5_)_6_]^−^ were so far unsuccessful. Instead, the solvent adduct [Xe(OTeF_5_)(SO_2_ClF)][Sb(OTeF_5_)_6_] was always obtained, indicating the near-linear alignment at the xenon centre.^11,12^ Furthermore, the [XeOTeF_5_]^+^ cation has been proved to be a strong two-electron oxidizer, which is *e.g.* a useful synthon for the preparation of trihalomethyl carbocations.^13^

Additionally, due to its chemical robustness, the teflate group has been utilized to prepare well-performing WCAs.^14^ Our group reported on the [Al(OTeF_5_)_4_]^−^ anion in 2017,^15^ which has subsequently allowed the stabilization of highly reactive cations, such as [P_4_H]^+^, [(CH_3_)_2_Cl]^+^, [(C_5_H_5_P)CH_3_]^+^ and [(C(C_6_F_5_)_3_)]^+^.^16–19^ Based on these achievements the teflate-based aluminate is a promising candidate for resisting the strong oxidizing property of a xenonium(ii) cation. The lack of N-donor adducts of the [XeOTeF_5_]^+^ cation, contrary to the well investigated ones for [XeF]^+^, prompted us to extend the chemistry of the teflate derivative, while pushing the limits of the [Al(OTeF_5_)_4_]^−^ anion to withstand strong oxidizing cations.

Herein we report on the formation and characterization of the cationic pentafluoroorthotellurato xenonium(ii) adducts with fluorinated pyridines, namely [Xe(OTeF_5_)(py^F^)]^+^ (py^F^ = C_5_F_5_N, C_5_H_3_F_2_N), as their salts of the weakly coordinating [Al(OTeF_5_)_4_]^−^ anion. Furthermore, the oxidizing properties of the cation [Xe(OTeF_5_)(C_5_F_5_N)]^+^ have been investigated. Moreover, the molecular structure of [Xe(OTeF_5_)(NC_5_F_5_)][Sb(OTeF_5_)_6_] was determined *via* single-crystal X-ray diffraction.

Based on the literature-known synthesis of the [FXe(NC_5_F_5_)]^+^ cation, starting from [HNC_5_F_5_][AsF_6_] and XeF_2_,^20^ Xe(OTeF_5_)_2_ was reacted with our recently prepared Brønsted acid [HNC_5_F_5_][Al(OTeF_5_)_4_].^21^ However, various attempts to obtain [Xe(OTeF_5_)(NC_5_F_5_)][Al(OTeF_5_)_4_] (1) with this method were unsuccessful, resulting in multiple teflate- and pentafluoropyridine-containing species. Unfortunately, no xenon-compound was observed by ^129^Xe NMR spectroscopy.

Alternatively, we performed the abstraction of a teflate group from Xe(OTeF_5_)_2_ with the Lewis superacid Al(OTeF_5_)_3_,^22^ whereby both the [Xe(OTeF_5_)]^+^ cation and the corresponding WCA, [Al(OTeF_5_)_4_]^−^, should be formed. The equimolar reaction of Xe(OTeF_5_)_2_ with Al(OTeF_5_)_3_ in SO_2_ClF at −50 °C successfully results in a non-isolable intermediate with a distinct yellow colour, presumably [Xe(OTeF_5_)][Al(OTeF_5_)_4_]. Characterization of this intermediate was not possible with the low-temperature spectroscopic measurements. By subsequent addition of one equivalent of a fluorinated pyridine (py^F^ = C_5_F_5_N, C_5_H_3_F_2_N), the cationic adducts [Xe(OTeF_5_)(NC_5_F_5_)]^+^ (1) and [Xe(OTeF_5_)(NC_5_H_3_F_2_)]^+^ (2) as salts of the [Al(OTeF_5_)_4_]^−^ anion are formed (Scheme 1).

**Scheme 1:**
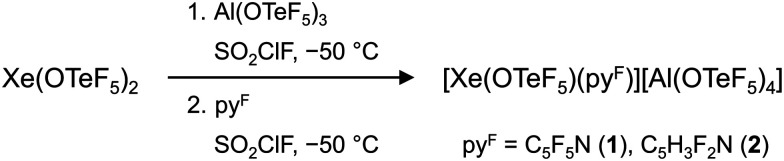
Synthesis of [Xe(OTeF_5_)(py^F^)][Al(OTeF_5_)_4_] (py^F^ = C_5_F_5_N, C_5_H_3_F_2_N).

The temperature-sensitive compounds 1 and 2 were characterized by NMR spectroscopy at −50 °C in SO_2_ClF, confirming the successful formation of the desired species (Table 1). Variable temperature NMR studies showed that compounds 1 and 2 decompose at temperatures higher than −10 °C. In the ^19^F NMR spectrum of 1, two magnetically inequivalent –OTeF_5_ groups with overlapping signals are observed. Simulation of the spectrum confirmed the expected two AB_4_ spin systems assigned to one teflate group in the cation and the four teflate groups located at the aluminium centre forming the WCA (Fig. 1a). The spectroscopic parameters of the latter are in agreement with those previously reported for the [Al(OTeF_5_)_4_]^−^ anion.^15^ The formation of this WCA to stabilize the xenonium(ii) cation is additionally proved by ^27^Al NMR spectra, as both adduct salts 1 and 2 show the characteristic resonance at *δ*(^27^Al) ≈ 46 ppm for the [Al(OTeF_5_)_4_]^−^ anion.

**Table 1:** ^19^F, ^27^Al and ^129^Xe NMR parameters for [Xe(OTeF_5_)(py^F^)][Al(OTeF_5_)_4_] (py^F^ = C_5_F_5_N, C_5_H_3_F_2_N)^a^

Species	Chemical shift (*δ*)^b^^c^ [ppm]							Coupling constant^b^ [Hz]		
	^19^F_A_	^19^F_B_	^129^Xe	^19^F_*o*_	^19^F_*m*_	^19^F_*p*_	^27^Al	^1^ *J*(^19^F_A_,^125^Te)	^2^ *J*(^19^F_A_,^19^F_B_)	^3^ *J*(^19^F_*o*_,^129^Xe)
[Xe(OTeF_5_)(NC_5_F_5_)]^+^	−45.4	−41.6	−2241	−86.7	−152.0	−108.3	—	3598	177	69
[Al(OTeF_5_)_4_]^−^	−38.4	−45.7	—	—	—	—	45.9	3353	188	—
[Xe(OTeF_5_)(NC_5_H_3_F_2_)]^+^	−43.1	−42.5	−2433	−67.6	—	—	—	3488	180	57
[Al(OTeF_5_)_4_]^−^	−38.1	−45.5	—	—	—	—	46.2	3338	186	—

a All NMR spectra were recorded in SO_2_ClF at −50 °C with [D_6_]acetone external locking.

b The ^19^F NMR data are reported according to the simulated spectra obtained with the gNMR software (see ESI).

c The symbols F_A_ and F_B_ denote equatorial and axial fluorine atoms, respectively, within the AB_4_ spin system of the –OTeF_5_ group. The symbols F_*o*_, F_*m*_, F_*p*_ represent *ortho*-, *meta*- and *para*-fluorine atoms of the fluorinated pyridine moieties.

**Fig. 1:**
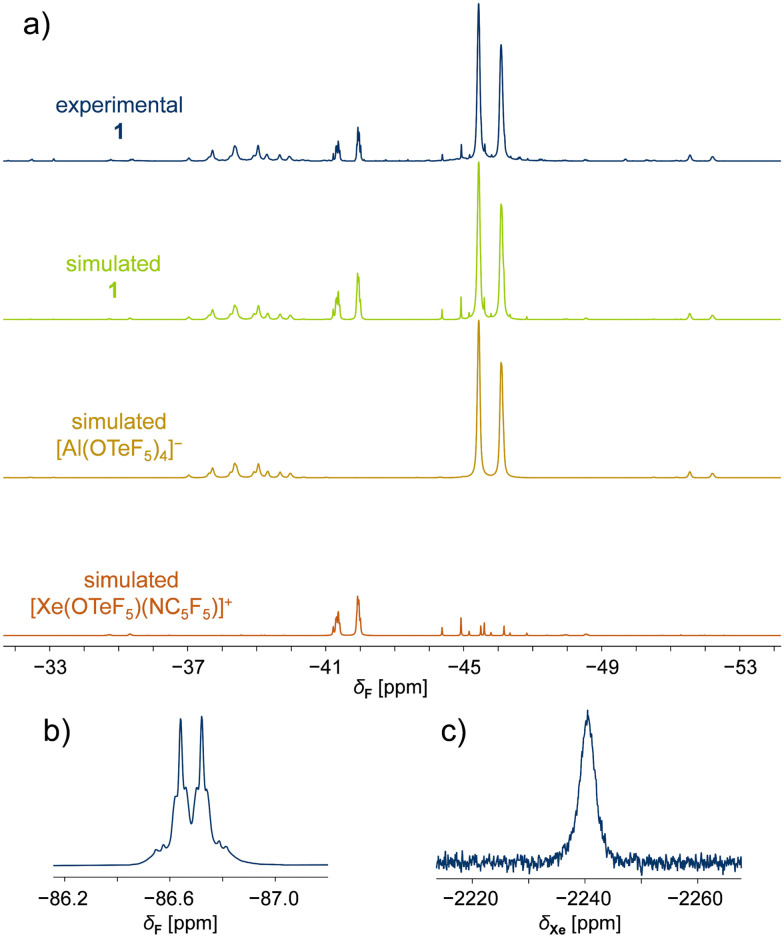
NMR spectra of [Xe(OTeF_5_)(NC_5_F_5_)][Al(OTeF_5_)_4_] (1) in SO_2_ClF ([D_6_]acetone (external lock), −50 °C). (a) ^19^F NMR (282 MHz) spectra showing the teflate region of (top to bottom) experimental 1, simulated 1, simulated [Al(OTeF_5_)_4_]^−^ anion and simulated [Xe(OTeF_5_)(NC_5_F_5_)]^+^ cation. (b) ^19^F NMR (282 MHz) spectrum of 1 depicting the *ortho* fluorine atoms of the pentafluoropyridine moiety, with the corresponding ^129^Xe satellites. (c) ^129^Xe NMR (83 MHz) spectrum of 1 depicting a broad singlet.

The AB_4_ pattern of the cations is inverted compared to the one of the [Al(OTeF_5_)_4_]^−^ anion, but in agreement with the observed ^19^F NMR spectrum of the previously reported [Xe(OTeF_5_)(SO_2_ClF)]^+^ cation.^12^ The chemical shifts of the [Xe(OTeF_5_)(NC_5_F_5_)]^+^ cation in 1 are found to be *δ*(^19^F_A_) = −45.4 ppm and *δ*(^19^F_B_) = −41.6 ppm, with the corresponding ^2^*J*(^19^F_A_,^19^F_B_) coupling constant of 177 Hz (Table 1). The resonances of the teflate group in the [Xe(OTeF_5_)(NC_5_H_3_F_2_)]^+^ cation in 2 are less separated, appearing at *δ*(^19^F_A_) = −43.1 ppm and *δ*(^19^F_B_) = −42.5 ppm, with the ^2^*J*(^19^F_A_,^19^F_B_) coupling constant being 180 Hz (Table 1).

Furthermore, the ^19^F NMR chemical shifts arising from the C_5_F_5_N moiety in the [Xe(OTeF_5_)(NC_5_F_5_)]^+^ cation in 1 (Table 1) are found to be downfield shifted with respect to those of neat C_5_F_5_N (Δ*δ*(^19^F_*ortho*_) = 3.0 ppm, Δ*δ*(^19^F_*meta*_) = 10.8 ppm, Δ*δ*(^19^F_*para*_) = 26.6 ppm). The *ortho*-fluorine resonance is accompanied by ^129^Xe (*I* = ½, 26.4%) satellites, arising from the ^3^*J*(^19^F,^129^Xe) spin–spin coupling (Fig. 1b). The presence of xenon satellites unambiguously demonstrates the coordination of C_5_F_5_N to the xenon(ii) centre. In the ^19^F NMR spectrum of 2, two different C_5_H_3_F_2_N species are observed. The signal corresponding to the cation of product 2 is easily identified, as it exhibits the characteristic ^129^Xe satellites with a ^3^*J*(^19^F,^129^Xe) coupling constant of 57 Hz. The secondary signal, resonating at a higher field, *δ*(^19^F_*ortho*_) = −77.8 ppm, than neat C_5_H_3_F_2_N is found to be [HNC_5_H_3_F_2_]^+^ cation by a control experiment.

The ^129^Xe NMR spectra of 1 and 2 consist of one xenon resonance appearing as a broad singlet at *δ*(^129^Xe) = −2241 ppm (Fig. 1c) and *δ*(^129^Xe) = −2433 ppm, respectively, indicating the presence of a single xenon species in each case. When compared to the previously reported [XeOTeF_5_]^+^ cation in SO_2_ClF at −50 °C, which shows a chemical shift of *δ*(^129^Xe) = −1459.5 ppm,^12^ the ^129^Xe chemical shifts of 1 and 2 are found to be shifted to lower frequencies upon adduct formation with nitrogen bases.

To further characterize our new xenonium cations, concentrated reaction mixtures of 1 and 2 were analysed by low-temperature Raman spectroscopy. The presence of strong fluorescence interference in the spectrum of 1 rendered its interpretation difficult. In the case of 2 (see Fig. S3.1 in ESI†), characteristic bands can be observed, which are in good agreement with the computed wavenumbers for the [Xe(OTeF_5_)(NC_5_H_3_F_2_)]^+^ cation at the B3LYP/def2-TZVPP level of theory. In particular, the prominent bands observed at *ν̃* = 140, 241 and 581 cm^−1^ are assigned to the stretching modes of O–Xe–N, Te–O–Xe and Xe–N, respectively. According to our experimental measurements, bands at *ν̃* = 303, 432 and 1221 cm^−1^ belong to remaining free SO_2_ClF in the sample. On the other hand, the Te–O and Te–F bands are overlapped with those from the [Al(OTeF_5_)_4_]^−^ anion, therefore hampering the assignment of the bands in the teflate region, 600–780 cm^−1^.

All our crystallization attempts for 1 and 2 to investigate the molecular structure of the new xenonium(ii) cationic adducts in the solid state were so far unsuccessful. Consequently, we changed the anion from [Al(OTeF_5_)_4_]^−^ to [Sb(OTeF_5_)_6_]^−^, which resulted useful to crystallize the [Xe(OTeF_5_)(SO_2_ClF)]^+^ cation.^11,12^ The salt [Xe(OTeF_5_)(NC_5_F_5_)][Sb(OTeF_5_)_6_] (3) was obtained by a similar procedure as 1 and 2. Namely, the salt [Xe(OTeF_5_)(SO_2_ClF)][Sb(OTeF_5_)_6_] was treated with one equivalent of C_5_F_5_N, forming a yellow solution. Characterization by multinuclear NMR spectroscopy showed that the ^19^F and ^129^Xe NMR data of 3 are in good agreement with those of the cation in the salt 1 (see ESI† for the complete data set). Unfortunately, the Raman spectrum of 3 shows strong fluorescence, which did not allow a detailed analysis, likewise in the case of 1.

Upon slowly cooling down the reaction mixture from −50 °C to −80 °C, single crystals of 3 suitable for X-ray diffraction could be obtained. Compound 3 crystallizes in the triclinic space group *P*1̄. The O1, Xe1 and N1 atoms of the cation are linearly aligned with an angle of 179.2(2)° (Fig. 2), which is consistent with the AX_2_E_3_ VSEPR molecular structure for a xenon(ii) species and closer to 180° than the previously reported O–Xe–O angle in the molecular structure of [Xe(OTeF_5_)(SO_2_ClF)][Sb(OTeF_5_)_6_] (174.2(2)°).^12^ The Te1–O1–Xe1 angle in 3 is 121.4(2)°, which is similar to the related SO_2_ClF adduct salt (120.8(2)°).^12^ The distance between the xenon and the oxygen atom of the teflate group in 3 is slightly elongated (Xe1–O1 = 202.8(4) pm), and the distance from the xenon to the nitrogen atom is shortened (Xe1–N1 = 233.4(5) pm), when compared with [Xe(OTeF_5_)(SO_2_ClF)]^+^ (Xe–O_teflate_ = 196.9(4) pm, Xe–O_solvent_ = 247.9(4) pm), and is in agreement with the higher basicity of C_5_F_5_N than of SO_2_ClF. The measured O1–Te1 distance of 188.3(5) pm is found to be in between the values reported for Xe(OTeF_5_)_2_ (184.3(11) pm)^10^ and the [Xe(OTeF_5_)(SO_2_ClF)]^+^ cation (193.8(5) pm).^12^

**Fig. 2:**
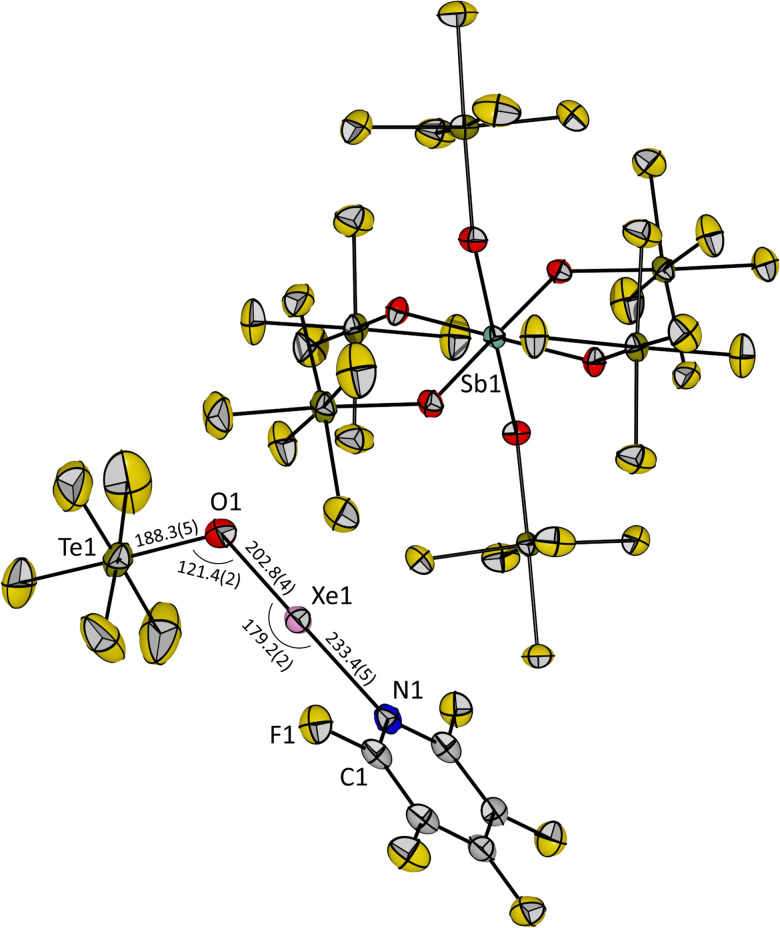
Molecular structure of [Xe(OTeF_5_)(NC_5_F_5_)][Sb(OTeF_5_)_6_] (3) in the solid state. Displacement ellipsoids set at 50% probability. Selected bond lengths [pm] and angles [°] are depicted. For crystallographic details see ESI.†

Finally, to demonstrate the oxidation potential of [Xe(OTeF_5_)(NC_5_F_5_)][Al(OTeF_5_)_4_] (1), the compound was reacted with an excess of tris(2,4,6-tribromophenyl)amine in order to oxidize it to the corresponding ammoniumyl radical cation, which is a variation of tris(4-tribromophenyl)amine (“magic blue”) with an even higher standard oxidation potential.^23^ An immediate colour change from brown to deep-purple with gas evolution was observed upon addition of the amine, indicating the formation of the tris(2,4,6-tribromophenyl)ammoniumyl radical cation. This was proved by EPR spectroscopy showing one broad signal with a *g* value of 2.009, assigned to the radical cation (see Fig. S5, ESI†). Furthermore, the oxidation potential of xenonium cations has been evaluated by adiabatic ionization energy calculations at the B3LYP/def2-TZVPP level of theory and resulted to be XeF/XeF^+^ 10.7, Xe(OTeF_5_)/[Xe(OTeF_5_)]^+^ 10.6, and Xe(OTeF_5_)(NC_5_F_5_)/[Xe(OTeF_5_)(NC_5_F_5_)]^+^ 9.2 eV (see also ESI,† Table S4).

In conclusion, we have demonstrated that the Lewis superacid Al(OTeF_5_)_3_ is able to abstract a teflate group from Xe(OTeF_5_)_2_ to presumably form the [Xe(OTeF_5_)]^+^ cation and the weakly coordinating [Al(OTeF_5_)_4_]^−^ anion. This intermediate could be subsequently stabilized upon coordination of the oxidation-resistant nitrogen bases C_5_F_5_N and C_5_H_3_F_2_N. This way, the [Xe(OTeF_5_)(py^F^)][Al(OTeF_5_)_4_] (py^F^ = C_5_F_5_N, C_5_H_3_F_2_N) salts were prepared and characterized by low-temperature NMR and Raman spectroscopy, entailing the first xenonium(ii) cations stabilized by the [Al(OTeF_5_)_4_]^−^ WCA. Also, the synthesis of the related [Sb(OTeF_5_)_6_]^−^ salt of the [Xe(OTeF_5_)(NC_5_F_5_)]^+^ cation enabled us to structurally characterize this unprecedented cation for the first time. Moreover, we have experimentally shown the high oxidation potential of the adduct salt [Xe(OTeF_5_)(NC_5_F_5_)][Al(OTeF_5_)_4_] (1), which oxidizes tris(2,4,6-tribromophenyl)amine to form the corresponding ammoniumyl radical cation.

This work was funded by the Deutsche Forschungsgemeinschaft (DFG, German Research Foundation) – Project ID: 387284271 (SFB 1349: Fluorine-Specific Interactions) and the European Research Council (ERC) – Project HighPotOx. A. P.-B. thanks the Fonds der Chemischen Industrie for a Liebig Fellowship. Computing resource was provided by the Zentrum für Elektronische Datenbearbeitung (ZEDAT) at Freie Universität Berlin. The authors thank the core facility Biosupramol for analytical measurements and acknowledge MSc Liza Richter (Humboldt-Universität zu Berlin), MSc Deniz Meyer (Freie Universität Berlin) and MSc Johanna Schlögl (Freie Universität Berlin) for performing spectroscopic measurements.

## Conflicts of interest

There are no conflicts to declare.

## Supplementary Material

CC-060-D3CC05560D-s001

CC-060-D3CC05560D-s002
