# Supplementary material for: [Xe(OTeF5)(pyF)]+: a strong oxidizing xenonium(ii) teflate cation with N-donor bases
Source: Chem Commun (Camb). 2023 Dec 13;60(13):1711–4. doi: 10.1039/d3cc05560d (PMC10852027; doi:10.1039/d3cc05560d)
Supplement: CC-060-D3CC05560D-s001 [file CC-060-D3CC05560D-s001.pdf]

Electronic Supplementary Material (ESI) for ChemComm.
This journal is © The Royal Society of Chemistry 2023

Supporting Information

for

[Xe(OTeF5)(pyF)]+: A Strong Oxidizing Xenonium(II) Teflate Cation with N-Donor Bases

Ahmet N. Toraman, Lukas Fischer, Alberto Pérez-Bitrián, Anja, Wiesner, Kurt F. Hoffmann and Sebastian Riedel

Table of Contents

1. Experimental Section 2
2. NMR Spectra 5
3. Raman Spectrum of $[Xe(OTeF_5)(NC_5H_3F_2)][Al(OTeF_5)_4]$ (2) 9
4. IR Spectra 10
5. EPR Spectrum 11
6. Crystal Data 12
7. Quantum Chemical Calculations 13
8. References 20

1. Experimental Section

1.1 General Procedures and Materials

All preparative work was performed using standard Schlenk techniques, ensuring the absence of moisture and oxygen. Reactions were carried out in greaseless Schlenk tubes with PTFE valves. Solids were handled in a *MBRAUN UNIlab plus* glovebox with an argon atmosphere (O_2 < 0.5 ppm, H_2O < 0.5 ppm). $Al(OTeF_5)_3$, $Sb(OTeF_5)_3$ and $Xe(OTeF_5)_2$ were prepared as described elsewhere.[1,2,3,4] SO_2ClF was freshly dried with CaH_2 before use. Pentafluoropyridine and 2,6-difluoropyridine were dried with CaH_2 and distilled over 3 Å molecular sieves before degassing. The NMR spectra were recorded on a *Bruker Avance III 300* spectrometer. All reported chemical shifts (δ in ppm) are referenced to the Ξ values as recommended by IUPAC.[5] Multiplicity is indicated as follows: s = singlet, d = doublet, dd = doublet of doublets, m = multiplet. Chemical shifts and coupling constants of strongly coupled spin systems in the ^{19}F NMR spectra are reported as simulated by *gNMR 5.0*.[6] Raman spectra were recorded at −196 °C on a *Bruker MultiRAM II* equipped with a low temperature Ge detector (1064 nm, 50–100 mW, resolution 4 cm^{-1}). IR spectra at −50 °C were recorded with a *Thermo Scientific Nicolet iS50 FTIR* (32 scans, resolution of 4 cm^{-1}) by using a previously published setup.[7] EPR spectrum was recorded with a *Magnettech MS 5000* spectrometer. Single crystal X-ray diffraction measurements were performed on a *Bruker D8 Venture* diffractometer using MoKα radiation with a CMOS area detector. Single crystals were picked under nitrogen atmosphere at −40 °C and mounted on a 0.15 mm *MiTeGen MicroMount* using perfluoroether oil. The crystal structure was solved with the *ShelXT* structure solution program[8] using intrinsic phasing and refined with the *ShelXL* refinement package[9] using the least-squares minimization by using the *OLEX2* software.[10] CCDC 2302485 contains the supplementary crystallographic data for this paper, which are provided free of charge by The Cambridge Crystallographic Data Centre. Crystal data and other details of the structure analyses are summarized in Table S1. Quantum chemical calculations were executed using *Turbomole V7.6.1* software,[11] employing the B3LYP functional[12] in conjugation with RI[13] and the def2-TZVPP basis set.[14] Normal mode analysis was used to characterize the minima on potential energy surfaces. Thermochemical data are given with zero-point energy correction derived from harmonic vibrational frequencies, without counterpoise correction. Elemental analysis could not be performed for any of the samples due to their temperature-sensitive nature and their strong oxidizing properties.

1.2 Synthesis of $[Xe(OTeF_5)(NC_5F_5)][Al(OTeF_5)_4]$ (1)

$Al(OTeF_5)_3$ (114.2 mg, 0.15 mmol, 1 equiv.) was dissolved in SO_2ClF (*ca.* 1 mL) at −50 °C. $Xe(OTeF_5)_2$ (93.6 mg, 0.15 mmol, 1 equiv.) was added to the stirring clear solution, resulting in an instant colour change to yellow. The mixture was stirred for 30 min at −50 °C and then C_5F_5N (26.0 mg, 0.15 mmol, 1 equiv.) was condensed onto the reaction mixture, forming a light brown solution. The nature of compound **1** was established by NMR and IR spectroscopy. Compound **1** decomposes when subjected to temperatures higher than −10 °C.

^{19}F NMR (282 MHz, SO_2ClF, ext. acetone-d$_6$, −50 °C, see Figure S2.1.1): δ = −38.4 (m, 1F_A, $^1J(^{19}F_A–^{125}Te)$ = 3350 Hz, Al–$OTeF_5$), −41.7 (m, 4F_B, $^2J(^{19}F_A–^{19}F_B)$ = 174, Xe–$OTeF_5$), −45.5 (m, 1F_A, $^1J(^{19}F_A–^{125}Te)$ = 3420 Hz, Xe–$OTeF_5$), −45.8 (m, 4F_B, $^2J(^{19}F_A–^{19}F_B)$ = 188 Hz, Al–$OTeF_5$), −86.7 (m, F_o, $^3J(^{19}F–^{129}Xe)$ = 68.7 Hz) −108.3 (m, F_p), −152.0 (m, 2F_m) ppm.
^{27}Al NMR (78 MHz, SO_2ClF, ext. acetone-d$_6$, −50 °C, see Figure S2.1.2): δ = 45.9 ppm (br, s).
^{129}Xe NMR (83 MHz, SO_2ClF, ext. acetone-d$_6$, −50 °C, see Figure S2.1.3): δ = −2240.5 ppm (br, s).
IR (ATR, −50 °C, see Figure S4.1): $\tilde{\nu}$ = 1657 (vw), 1542 (w), 1522 (m), 1444 (m), 1418 (vw), 1326 (vw), 1219 (m), 1112 (m), 1000 (w), 934 (m), 826 (m), 692 (vs), 655 (m), 624 (s), 595 (m), 549 (m), 491 (m), 298 (m), 166 (w) cm^{-1}.

1.3 Synthesis of $[Xe(OTeF_5)(NC_5H_3F_2)][Al(OTeF_5)_4]$ (2)

$Al(OTeF_5)_3$ (155.5 mg, 0.21 mmol, 1 equiv.) was dissolved in SO_2ClF (*ca.* 1 mL) at −50 °C. $Xe(OTeF_5)_2$ (127.4 mg, 0.21 mmol, 1 equiv.) was added to the stirring clear solution, resulting in an instant colour change to yellow. The mixture was stirred for 30 min at −50 °C and then $C_5H_3F_2N$ (24.1 mg, 0.21 mmol, 1 equiv.) was condensed onto the reaction mixture, forming a light green solution. The reaction mixture was analysed by NMR, IR and Raman spectroscopy. Compound **2** decomposes when subjected to temperatures higher than −10 °C. (The secondary $C_5H_3F_2N$ species was identified to be the $[HNC_5H_3F_2]^+$ cation by a control experiment, in which $C_5H_3F_2N$ was protonated by *o*DFB based Brønsted acid $[ArH][Al(OTeF_5)_4]$ (Ar = 1,2-difluorobenzene) at −30 °C in *o*DFB, as described elsewhere.[15])

^{1}H NMR (300 MHz, SO_2ClF, ext. acetone-d$_6$, −50 °C, see Figure S2.2.1): δ = 6.7 (d, 2H_m, $^3J(^1H–^{19}F)$ = 8.4 Hz), 6.8 (d, H_m, $^3J(^1H–^{19}F)$ = 8.2 Hz, $[HNC_5H_3F_2]^+$), 7.8 (m, 1H_p, $^3J(^1H–^1H)$ = 7.4 Hz), 8.0 (m, H_p, $^3J(^1H–^1H)$ = 7.7 Hz, $[HNC_5H_3F_2]^+$), 11.0 (s, $[HNC_5H_3F_2]^+$) ppm.
^{19}F NMR (282 MHz, SO_2ClF, ext. acetone-d$_6$, −50 °C, see Figure S2.2.2): δ = −38.2 (m, 1F_A, $^1J(^{19}F_A–^{125}Te)$ = 3420 Hz, Al–$OTeF_5$), −44.7 (d, 4F_B, $^2J(^{19}F_A–^{19}F_B)$ = 180, Xe–$OTeF_5$),

−45.2 (m, $1F_A$, $^{1}J(^{19}F_A–^{125}Te)$ = 3385 Hz, Xe–$OTeF_5$), −45.6 (d, $4F_B$, $^{2}J(^{19}F_A–^{19}F_B)$ = 186 Hz, Al–$OTeF_5$), −67.6 (dd, $2F_o$, $^{3}J(^{19}F–^{129}Xe)$ = 55.6 Hz), −77.8 (d, F_o, $[HNC_5H_3F_2]^+$) ppm.

^{27}Al NMR (78 MHz, SO_2ClF, ext. acetone-d_6, −50 °C, see Figure S2.2.3): δ = 46.2 ppm (br, s).

^{129}Xe NMR (83 MHz, SO_2ClF, ext. acetone-d_6, −50 °C, see Figure S2.2.4): δ = −2433.4 ppm (br, s).

IR (ATR, −50 °C, see Figure S4.2): $\tilde{\nu}$ = 1631 (w), 1489 (w), 1445 (w), 1278 (vw), 1220 (w), 1022 (vw), 933 (m), 829 (w), 688 (vs), 624 (s), 556 (m), 502 (w), 476 (w), 301 (m), 175 (w) cm^{-1}.

Raman (1064 nm, −196 °C, see Figure S3.1.1 and Figure S3.1.2): $\tilde{\nu}$ = 3213 (w), 2837 (w), 1348 (w), 1221 (w), 1076 (w), 1024 (w), 744 (sh, w), 734 (m), 694 (s), 650 (s), 581 (m), 504 (w), 472 (s), 454 (m), 432 (m), 375 (m), 332 (w), 303 (m), 241 (m), 140 (vs), 106 (m), 92 (s), 75 (s) cm^{-1}.

1.4 Synthesis of $[Xe(OTeF_5)(NC_5F_5)][Sb(OTeF_5)_6]$ (3)

By using a similar procedure for that used for $[Xe(OTeF_5)(SO_2ClF)][Sb(OTeF_5)_6]$,[16] this reaction was performed starting from dissolving $Sb(OTeF_5)_3$ (98.1 mg, 0.12 mmol, 1 equiv.) in SO_2ClF (*ca.* 1 mL) at −50 °C. $Xe(OTeF_5)_2$ (142.5 mg, 0.23 mmol, 2 equiv.) was added to the stirring cloudy mixture, resulting in a colour change to yellow after 30 min at −50 °C. After stirring for additional 30 min, C_5F_5N (19.8 mg, 0.12 mmol, 1 equiv.) was condensed onto the reaction mixture and reacted for 45 min, forming a yellow to brown coloured solution. Single crystals were obtained upon slowly cooling the concentrated solution from −50 °C to −80 °C.

^{19}F NMR (282 MHz, SO_2ClF, ext. acetone-d_6, −50 °C, see Figure S2.3.1): δ = −41.4 (m, $F_A \approx F_B$, $^{1}J(^{19}F_A–^{125}Te)$ = 3559 Hz, Sb–$OTeF_5$), −41.4 (m, $4F_B$, $^{2}J(^{19}F_A–^{19}F_B)$ = 179, Xe–$OTeF_5$), −45.2 (m, $1F_A$, $^{1}J(^{19}F_A–^{125}Te)$ = 3468 Hz, Xe–$OTeF_5$), −86.6 (m, F_o, $^{3}J(^{19}F–^{129}Xe)$ = 68.1 Hz) −108.1 (m, F_p), −151.8 (m, $2F_m$) ppm.

^{129}Xe NMR (83 MHz, SO_2ClF, ext. acetone-d_6, −50 °C, see Figure S2.3.2): δ = −2240.4 ppm (br, s).

1.5 Reaction of $[Xe(OTeF_5)(NC_5F_5)][Al(OTeF_5)_4]$ (1) with tris(2,4,6-tribromophenyl)amine

A solution of $[Xe(OTeF_5)(NC_5F_5)][Al(OTeF_5)_4]$ (**1**) in SO_2ClF (0.17 mmol/mL) at −50 °C was treated with solid tris(2,4,6-tribromophenyl)amine (390.6 mg, 0.41 mmol, 2.4 equiv.) Upon addition, the solution immediately changed colour to deep purple and gas evolution was observed. Volatile was removed *in vacuo* while warming up the flask to the ambient temperature, then the remaining was dissolved in 1 mL CH_2Cl_2. The mixture was analysed by EPR spectroscopy at room temperature (see Figure S5).

2. NMR Spectra

2.1. NMR Spectra of [Xe(OTeF$_5$)(NC$_5$F$_5$)][Al(OTeF$_5$)$_4$] (1)

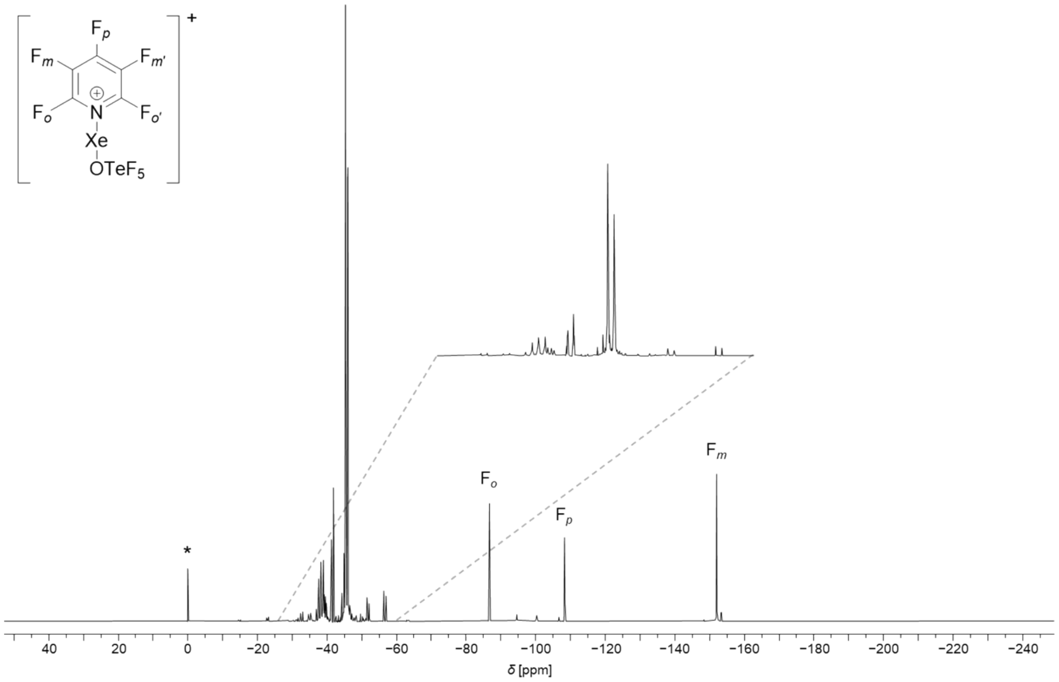

Figure S2.1.1. ^{19}F NMR (282 MHz, SO$_2$ClF, ext. acetone-d$_6$, −50 °C) spectrum of [Xe(OTeF$_5$)(NC$_5$F$_5$)][Al(OTeF$_5$)$_4$] (**1**) (*: ext. CFCl$_3$).

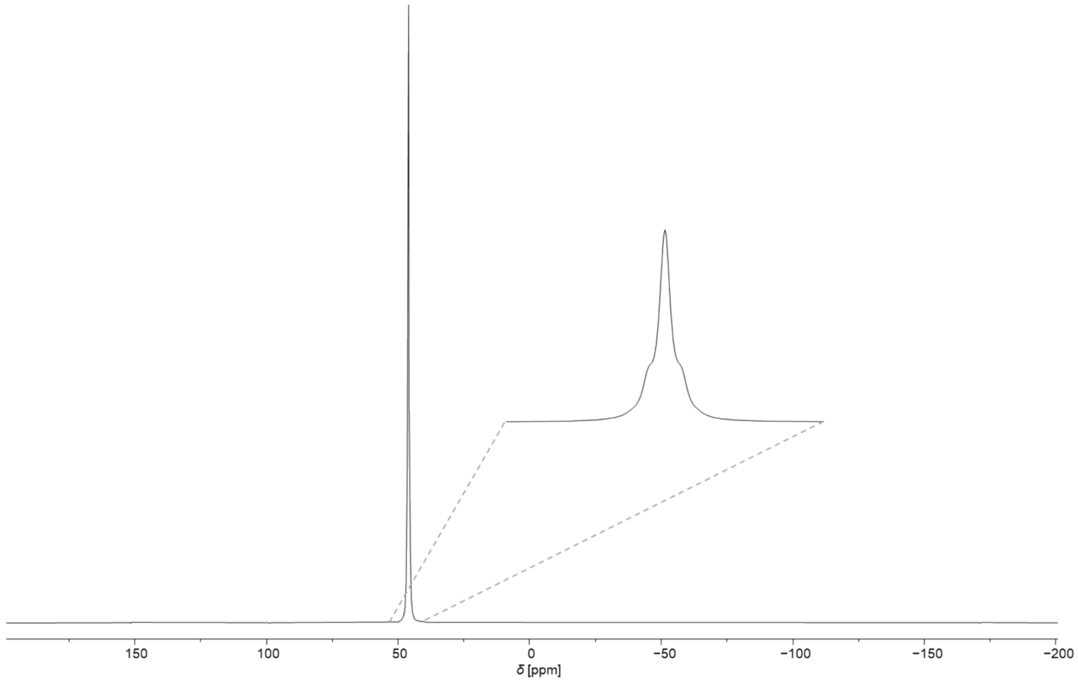

Figure S2.1.2. ^{27}Al NMR (78 MHz, SO$_2$ClF, ext. acetone-d$_6$, −50 °C) spectrum of [Xe(OTeF$_5$)(NC$_5$F$_5$)][Al(OTeF$_5$)$_4$] (**1**).

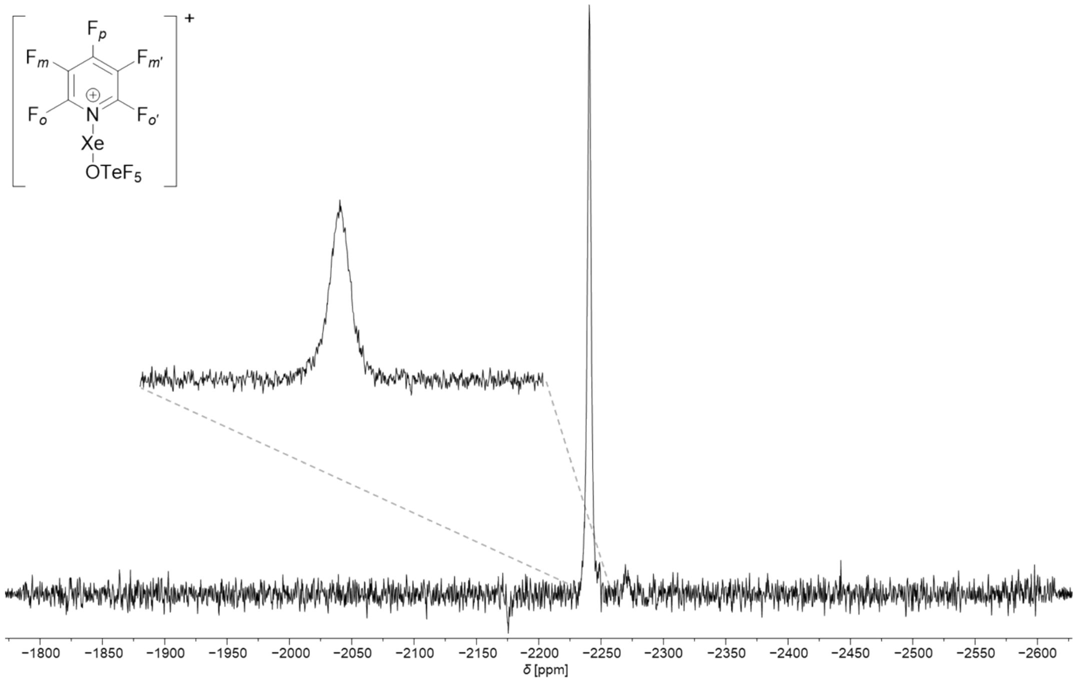

Figure S2.1.3. ^{129}Xe NMR (83 MHz, SO_2ClF, ext. acetone-d_6, −50 °C) spectrum of $[Xe(OTeF_5)(NC_5F_5)][Al(OTeF_5)_4]$ (**1**).

2.2. NMR Spectra of $[Xe(OTeF_5)(NC_5H_3F_2)][Al(OTeF_5)_4]$ (2)

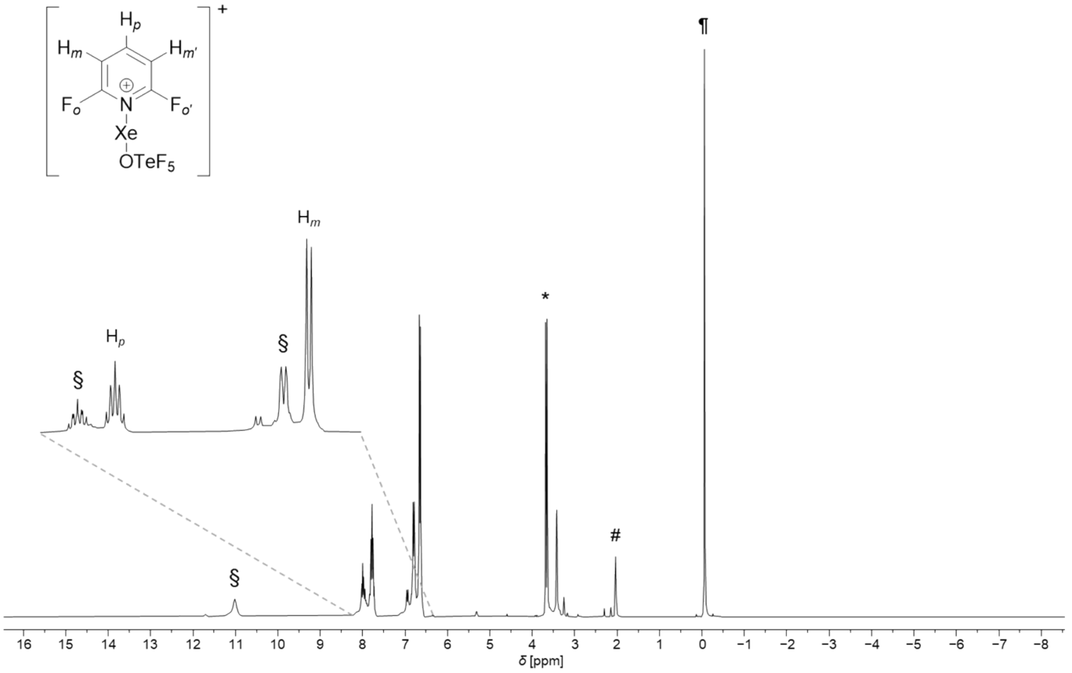

Figure S2.2.1. ^{1}H NMR (300 MHz, SO_2ClF, ext. acetone-d_6, −50 °C) spectrum of $[Xe(OTeF_5)(NC_5H_3F_2)][Al(OTeF_5)_4]$ (**2**) (¶: ext. $Si(CH_3)_4$, #: ext. $(CD_3)_2CO$ *: ext. $(CH_3O)_3PO$, §: $[HC_5H_3F_2N]^+$ cation).

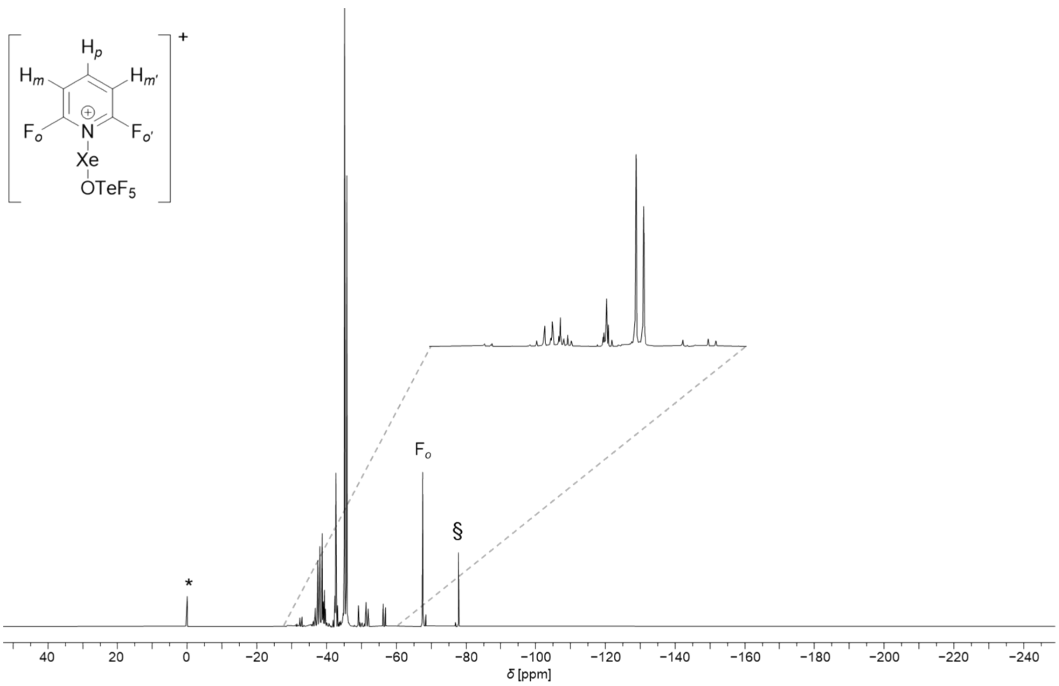

Figure S2.2.2. ^{19}F NMR (282 MHz, SO_2ClF, ext. acetone-d_6, −50 °C) spectrum of $[Xe(OTeF_5)(NC_5H_3F_2)][Al(OTeF_5)_4]$ (**2**) (*: ext. $CFCl_3$, §: $[HC_5H_3F_2N]^+$ cation).

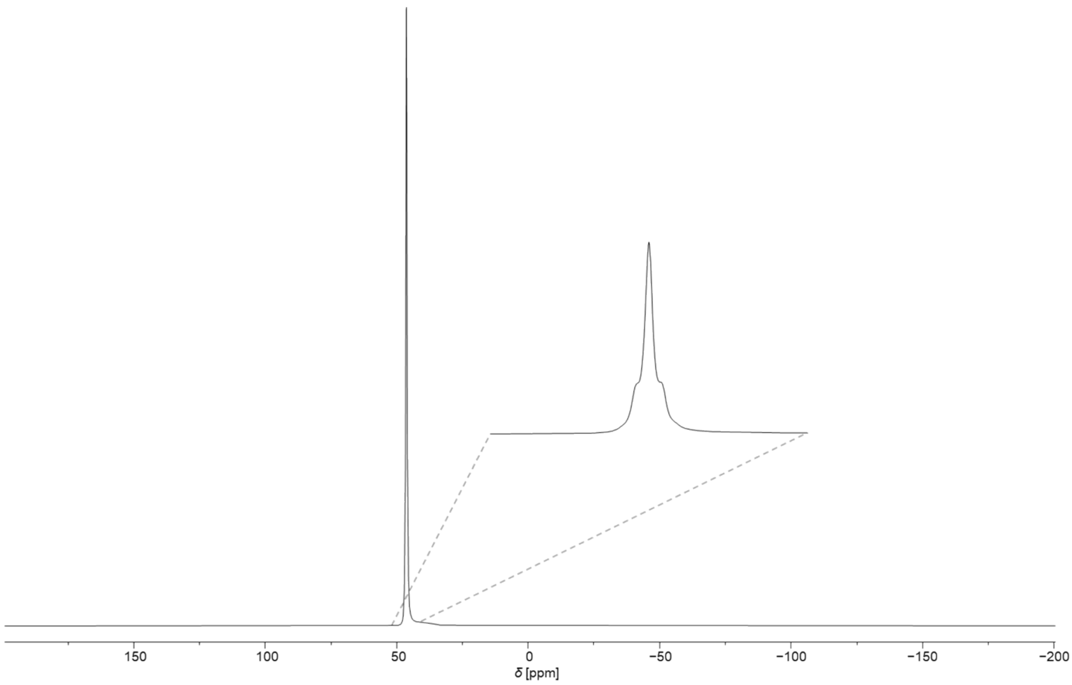

Figure S2.2.3. ^{27}Al NMR (78 MHz, SO_2ClF, ext. acetone-d_6, −50 °C) spectrum of $[Xe(OTeF_5)(NC_5H_3F_2)][Al(OTeF_5)_4]$ (**2**).

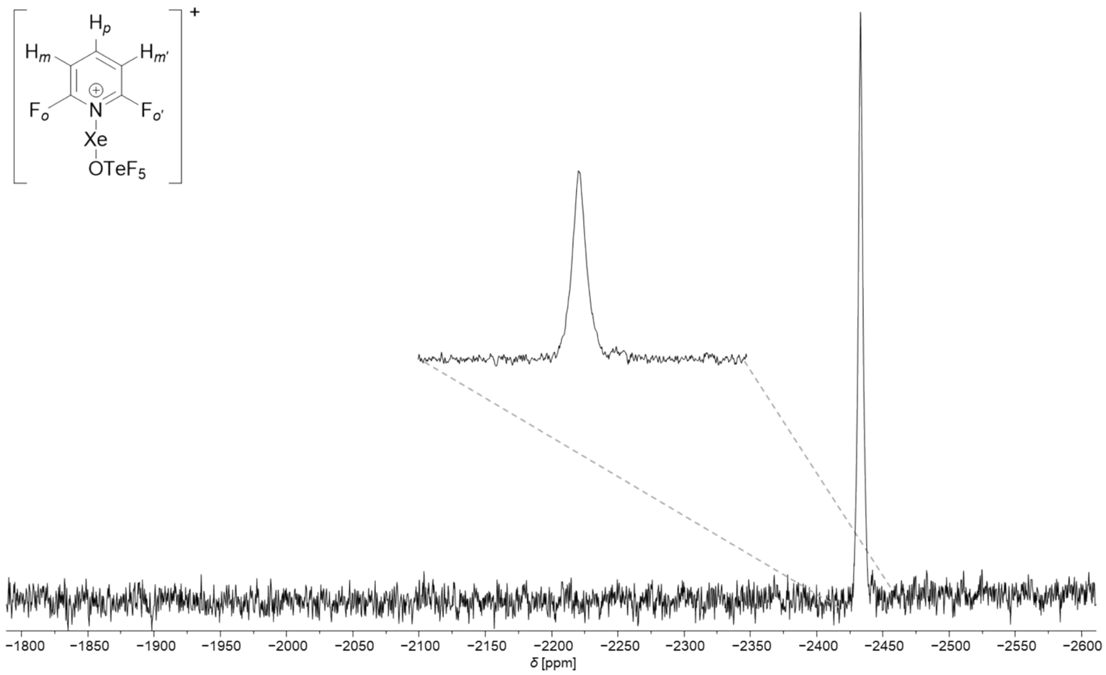

Figure S2.2.4. ^{129}Xe NMR (83 MHz, SO_2ClF, ext. acetone-d_6, −50 °C) spectrum of $[Xe(OTeF_5)(NC_5H_3F_2)][Al(OTeF_5)_4]$ (**2**).

2.3. NMR Spectra of $[Xe(OTeF_5)(NC_5F_5)][Sb(OTeF_5)_6]$ (3)

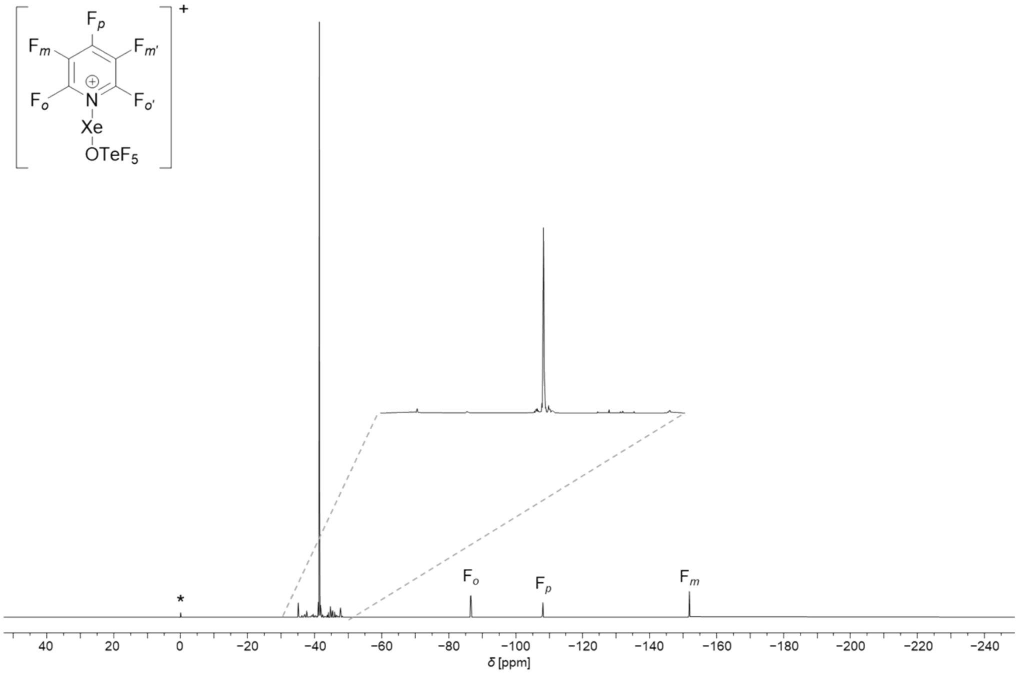

Figure S2.3.1. ^{19}F NMR (282 MHz, SO_2ClF, ext. acetone-d_6, −50 °C) spectrum of $[Xe(OTeF_5)(NC_5F_5)][Sb(OTeF_5)_6]$ (**3**) (*: ext. $CFCl_3$).

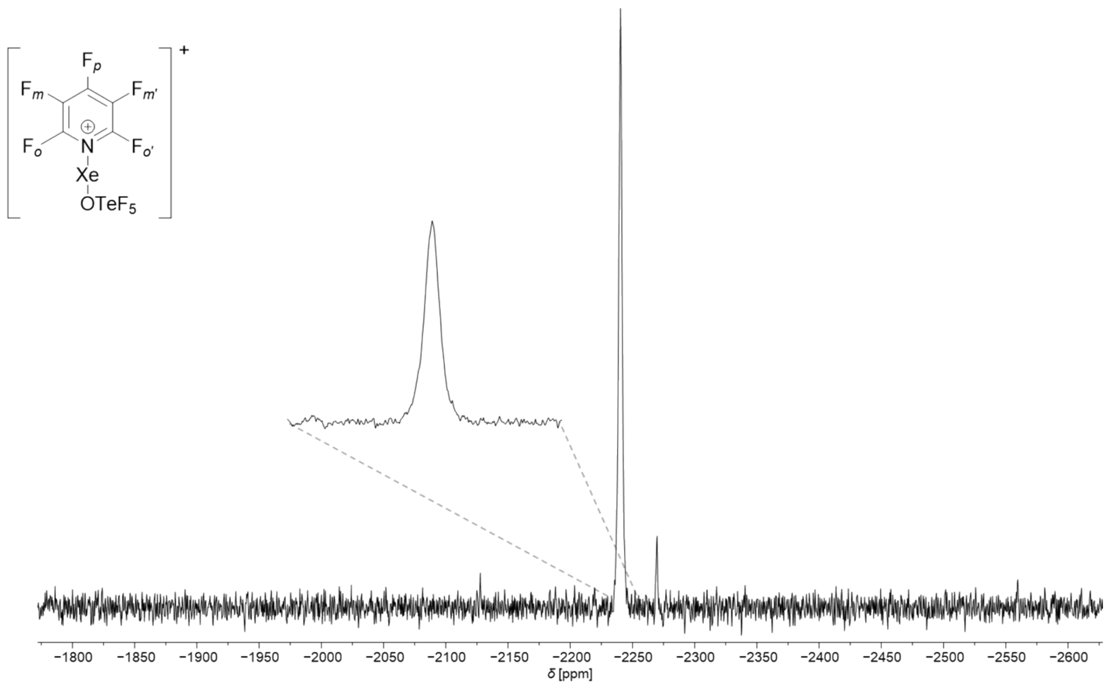

Figure S2.3.2. ^{129}Xe NMR (83 MHz, SO_2ClF, ext. acetone-d_6, −50 °C) spectrum of $[Xe(OTeF_5)(NC_5F_5)][Sb(OTeF_5)_6]$ (**3**).

3. Raman Spectrum of $[Xe(OTeF_5)(NC_5H_3F_2)][Al(OTeF_5)_4]$ (2)

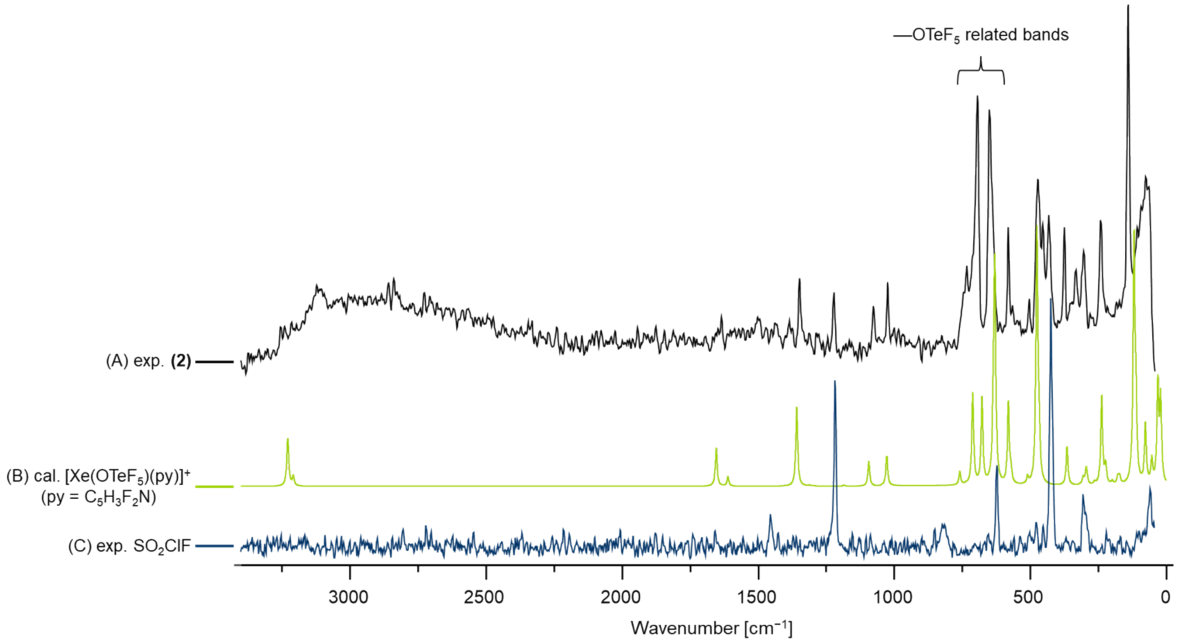

Figure S3.1. (A) Experimental Raman spectrum of $[Xe(OTeF_5)(NC_5H_3F_2)][Al(OTeF_5)_4]$ (**2**) at −196 °C. (B) Calculated Raman spectrum of $[Xe(OTeF_5)(NC_5H_3F_2)]^+$ at B3LYP/def2-TZVPP level of theory. (C) Experimental Raman spectrum of SO_2ClF at −196 °C.

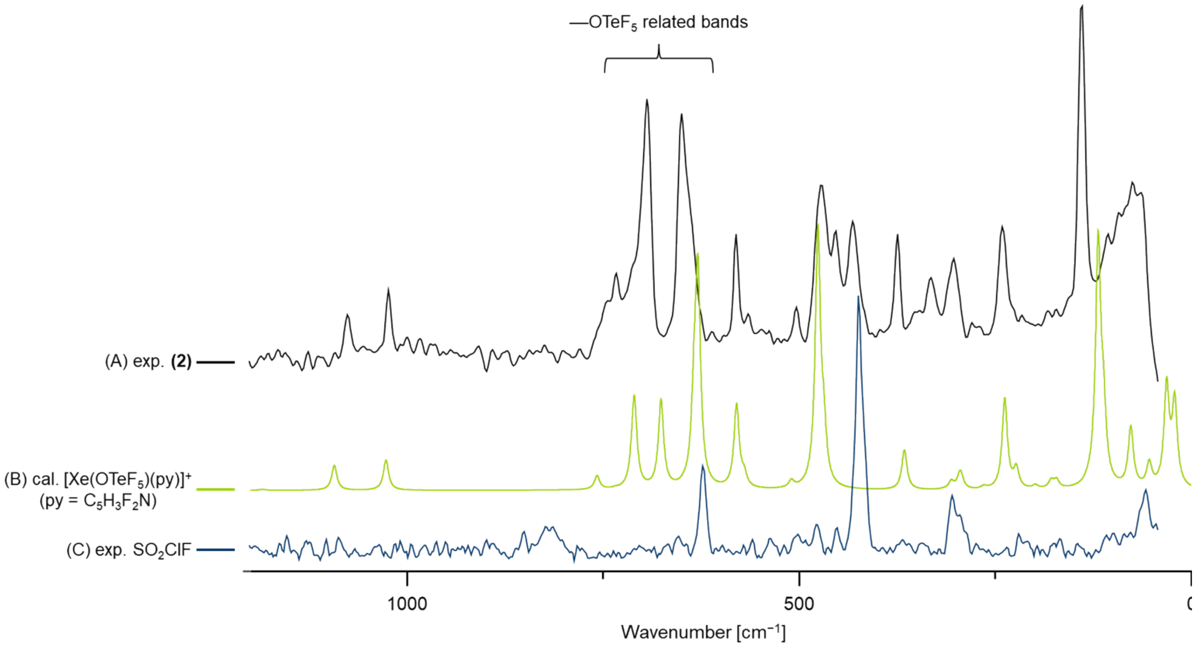

Figure S3.2. (A) Enlarged experimental Raman spectrum of $[Xe(OTeF_5)(NC_5H_3F_2)][Al(OTeF_5)_4]$ (**2**) at −196 °C. (B) Calculated Raman spectrum of $[Xe(OTeF_5)(NC_5H_3F_2)]^+$ at B3LYP/def2-TZVPP level of theory. (C) Experimental Raman spectrum of SO_2ClF at −196 °C.

4. IR Spectra

4.1. IR Spectrum of $[Xe(OTeF_5)(NC_5F_5)][Al(OTeF_5)_4]$ (1)

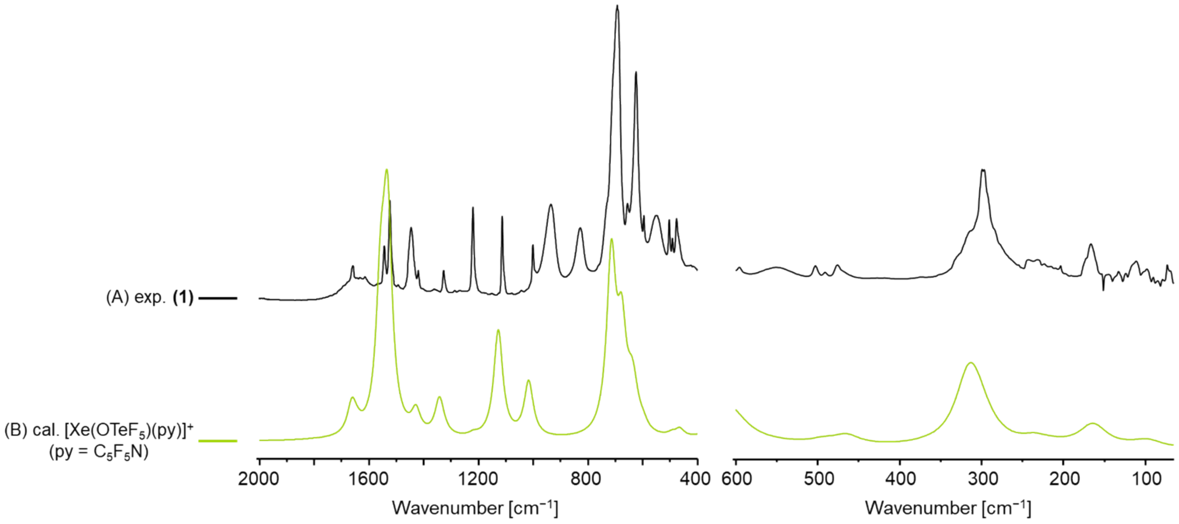

Figure S4.1. (A) Experimental IR spectrum of $[Xe(OTeF_5)(NC_5F_5)][Al(OTeF_5)_4]$ (**1**) at −50 °C (ATR). (B) Calculated IR spectrum of $[Xe(OTeF_5)(NC_5F_5)]^+$ at B3LYP/def2-TZVPP level of theory (Left: mid-IR, Right: far-IR).

4.2. IR Spectrum of [Xe(OTeF$_5$)(NC$_5$H$_3$F$_2$)][Al(OTeF$_5$)$_4$] (2)

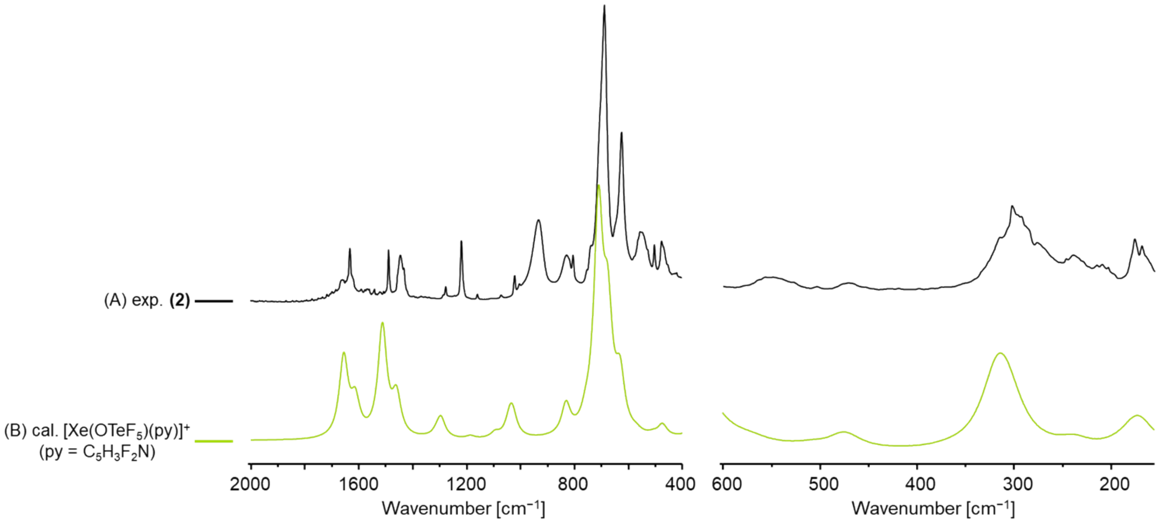

Figure S4.2. (A) Experimental IR spectrum of [Xe(OTeF$_5$)(NC$_5$H$_3$F$_2$)][Al(OTeF$_5$)$_4$] (**2**) at −50 °C (ATR). (B) Calculated IR spectrum of [Xe(OTeF$_5$)(NC$_5$H$_3$F$_2$)]$^+$ at B3LYP/def2-TZVPP level of theory (Left: mid-IR, Right: far-IR).

5. EPR Spectrum

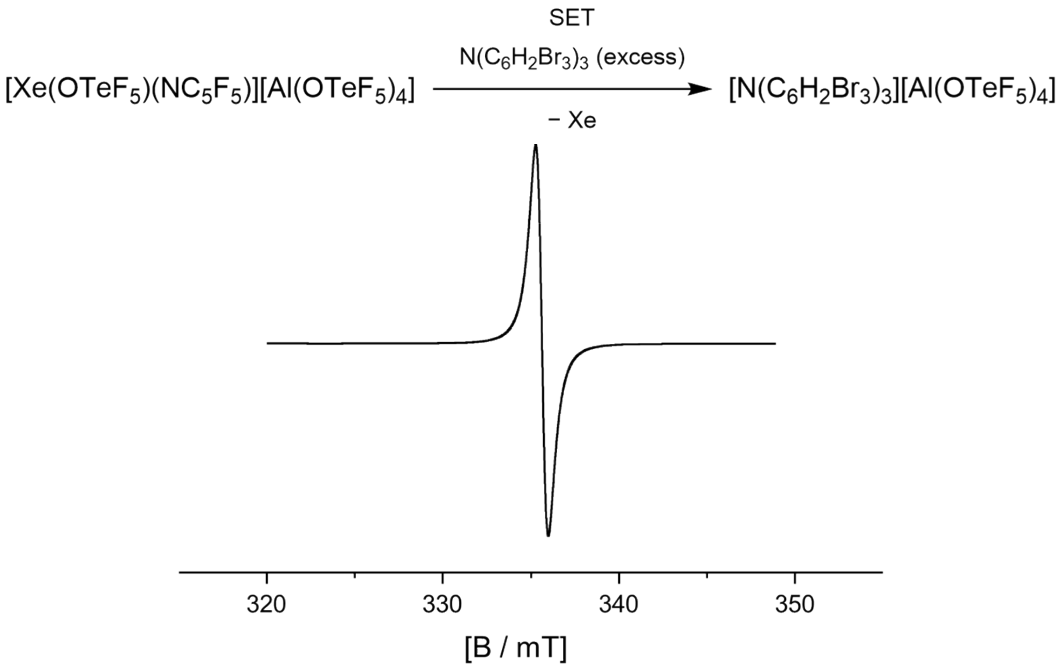

Figure S5. Experimental EPR spectrum showing the signal of the ammoniumyl radical cation [N(C$_6$H$_2$Br-2,4,6)$_3$]$^{\bullet+}$ at room temperature. The spectrum was recorded after the reaction of [Xe(OTeF$_5$)(NC$_5$F$_5$)][Al(OTeF$_5$)$_4$] (**1**) with N(C$_6$H$_2$Br-2,4,6)$_3$ in CH$_2$Cl$_2$ at room temperature, according to the shown equation.

6. Crystal Data

Table S1. Crystal data and structure refinement for compound [Xe(OTeF$_5$)(NC$_5$F$_5$)][Sb(OTeF$_5$)$_6$] (**3**).

Empirical formula	$C_5Cl_{3.4}F_{43.4}NO_{13.8}S_{3.4}SbTe_7Xe$
Formula weight	2495.24
Temperature/K	150.0
Crystal system	triclinic
Space group	$P\bar{1}$
a [Å]	9.9625(5)
b [Å]	13.7996(12)
c [Å]	20.0668(18)
α [°]	102.386(2)
β [°]	95.157(2)
γ [°]	93.654(2)
Volume [Å^3]	2673.8(4)
Z	2
ρ_{calc} [g·cm^3]	3.099
μ [mm^{-1}]	5.382
F(000)	2238.0
Crystal size [mm^3]	0.551 × 0.502 × 0.318
Radiation	MoKα (λ = 0.71073)
2Θ range for data collection [°]	4.046 to 52.956
Index ranges	$-12 \leq h \leq 11, -17 \leq k \leq 17, -25 \leq l \leq 25$
Reflections collected	87933
Independent reflections	10993 [R_{int} = 0.0513, R_{sigma} = 0.0299]
Data/Restraints/Parameters	10993/1034/847
Goodness-of-fit on F^2	1.139
Final *R* indexes [I>=2σ (I)]	R_1 = 0.0326, wR_2 = 0.0849
Final *R* indexes [all data]	R_1 = 0.0347, wR_2 = 0.0861
Largest diff. peak/hole [eÅ^{-3}]	1.58/−1.18
CCDC number	2302485

7. Quantum Chemical Calculations

Table S2 Selected experimental [Xe(OTeF$_5$)(NC$_5$F$_5$)][Sb(OTeF$_5$)$_6$] (**3**) and calculated (B3LYP/def2-TZVPP) geometrical parameters for the [Xe(OTeF$_5$)(NC$_5$F$_5$)]$^+$ cationic adduct.

	Bond lengths [pm]			Bond angles [°]	
	Exp.	Calc.		Exp.	Calc.
Xe1–N1	233.4(5)	243.2	Xe1–N1–C1	119.6(4)	120.0
O1–X1	202.8(4)	202.0	O1–Xe1–N1	179.2(2)	175.9
Te1–O1	188.3(5)	195.3	Te1–O1–Xe1	121.4(2)	121.5
N1–C1	133.2(8)	133.1	N1–C1–F1	116.0(5)	116.9
C1–F1	131.6(7)	131.5			

Table S3 Calculated reaction enthalpies for the formation of [Xe(OTeF$_5$)(py^F)]$^+$ (py^F = C$_5$F$_5$N, C$_5$H$_3$F$_2$N) adducts

	ΔH^a [kJ mol^{-1}]
[Xe(OTeF$_5$)]$^+$ + C$_5$F$_5$N → [Xe(OTeF$_5$)(NC$_5$F$_5$)]$^+$	−139.9
[Xe(OTeF$_5$)]$^+$ + C$_5$H$_3$F$_2$N→ [Xe(OTeF$_5$)(NC$_5$H$_3$F$_2$)]$^+$	−188.8

[a] Calculations were performed on B3LYP/def2-TZVPP level of theory, neglecting interactions with the weakly coordinating [Al(OTeF$_5$)$_4$]$^-$ anion.

Table S4 Calculated adiabatic ionization energies[a], literature known experimental ionization potentials and experimental oxidation potential for selected compounds.

	$E_{calc}{}^a$ [eV]	E_{exp} [eV]	E° [V]
XeF → [XeF]$^+$	10.71	10.23[17], 10.3[18]	
Xe(OTeF$_5$) → [Xe(OTeF$_5$)]$^+$	10.55		
Xe(OTeF$_5$)(NC$_5$F$_5$)→ [Xe(OTeF$_5$)(NC$_5$F$_5$)]$^+$	9.22		
FXe(NC$_5$F$_5$) → [FXe(NC$_5$F$_5$)]$^+$	8.82		
N(C$_6$Br$_3$H$_2$)$_3$ → [N(C$_6$Br$_3$H$_2$)$_3$]$^+$	7.31		1.96[19]

[a] Calculations were performed on B3LYP/def2-TZVPP level of theory.

Structure optimizations were performed using the B3LYP functional together with RI, D4 dispersion correction and def2-TZVPP basis set as implemented in the Turbomole V7.6.1 program.

7.1 XeF (neutral)
7.2 $[XeF]^+$
7.3 $Xe(OTeF_5)$ (neutral)
7.4 $[Xe(OTeF_5)]^+$
7.5 $Xe(OTeF_5)(NC_5F_5)$ (neutral)
7.6 $[Xe(OTeF_5)(NC_5F_5)]^+$
7.7 $FXe(NC_5F_5)$ (neutral)
7.8 $[FXe(NC_5F_5)]^+$
7.9 $N(C_6H_2Br_3)_3$
7.10 $[N(C_6H_2Br_3)_3]^+$
7.11 C_5F_5N
7.12 $C_5H_3F_2N$
7.13 $[Xe(OTeF_5)(NC_5H_3F_2)]^+$

7.1 XeF (neutral)

```
$coord
   0.00000000000000    0.00000000000000   -2.26409848125068  xe
   0.00000000000000    0.00000000000000    2.26409848125068  f
$end
```

E_{tot} = -429.1207424198 H

7.2 $[XeF]^+$

```
$coord
   0.00000000000000    0.00000000000000   -1.77340422409758  xe
   0.00000000000000    0.00000000000000    1.77340422409758  f
$end
```

E_{tot} = -428.7282369377 H

7.3 $Xe(OTeF_5)$ (neutral)

```
$coord
   3.64038128444577    4.99188079524722    0.33441697040895  xe
   8.07403152005769    5.05942368337035   -2.20556760708574  o
  10.97359852108073    5.16112722054465   -0.06319585569100  te
  13.83452219100956    5.25629180296729    1.94709348470183  f
  12.38973083610329    7.69633034290789   -2.04311634611858  f
   9.53354273795706    7.58181559924671    2.05302613076988  f
   9.71266932442594    2.63219064502057    2.03985642502644  f
  12.56453906364672    2.74763555578673   -2.06249430475048  f
$end
```

E_{tot} = -1171.823564596 H

7.4 $[Xe(OTeF_5)]^+$

```
$coord
   4.48412558820094    5.00588392894357   -0.23194050777019  xe
   7.78145952139043    5.05624143243056   -1.92168678155418  o
  11.04200386958330    5.16506590050482    0.11485749128165  te
  14.00854060764404    5.26387705578176    1.85943342248510  f
  11.99793687116185    7.66705952164483   -2.08468443892479  f
   9.53838119725083    7.53913532864350    2.19664830985069  f
   9.71086883008722    2.67557090158005    2.17678096694912  f
  12.15969899340815    2.75386157556233   -2.10938956505607  f
$end
```

E_{tot} = -1171.436763682 H

7.5 $Xe(OTeF_5)(NC_5F_5)$ (neutral)

```
$coord
  -3.90609743625420   -0.24750914565435   -1.37405883017776  xe
  -0.36782861589140   -1.30983481355048   -2.33444507146070  o
   2.11188023517464   -2.26576266185521    0.21453897263901  te
   4.45122273914070   -3.16128610409932    2.60640234565488  f
  -0.38503553613140   -4.03602643488527    1.95095972609762  f
   2.83872250864019   -5.09407235143956   -1.67642464099891  f
   1.23823775022559    0.57743057664385    2.09108356606981  f
   4.48037405688525   -0.42583591392805   -1.54566314841434  f
  -8.23169641727171    1.05925339805429   -0.53679283118141  n
  -8.87883941853383    3.48004266369633   -0.74917294832000  c
  -9.94970996567712   -0.65155936061869    0.13114753836348  c
 -12.43420226127043   -0.00967496648577    0.62353554960226  c
 -13.12021433027111    2.52623549691182    0.40177405580418  c
 -11.31346461600294    4.31431629079931   -0.29776165230253  c
  -9.17940750954931   -3.00725443644106    0.30494723399283  f
 -14.09790330352151   -1.73341865777035    1.27945213492892  f
 -15.44382202512921    3.22330018267177    0.84697759908452  f
 -11.90813815068515    6.71561173327124   -0.52071965594901  f
  -7.08651157469176    5.06647992191678   -1.41574214891018  f
$end
```

E_{tot} = -1915.969171034 H

7.6 $[Xe(OTeF_5)(NC_5F_5)]^+$

```
$coord
  -3.90609743625420   -0.24750914565435   -1.37405883017776  xe
  -0.36782861589140   -1.30983481355048   -2.33444507146070  o
   2.11188023517464   -2.26576266185521    0.21453897263901  te
   4.45122273914070   -3.16128610409932    2.60640234565488  f
  -0.38503553613140   -4.03602643488527    1.95095972609762  f
   2.83872250864019   -5.09407235143956   -1.67642464099891  f
   1.23823775022559    0.57743057664385    2.09108356606981  f
   4.48037405688525   -0.42583591392805   -1.54566314841434  f
  -8.23169641727171    1.05925339805429   -0.53679283118141  n
  -8.87883941853383    3.48004266369633   -0.74917294832000  c
  -9.94970996567712   -0.65155936061869    0.13114753836348  c
 -12.43420226127043   -0.00967496648577    0.62353554960226  c
 -13.12021433027111    2.52623549691182    0.40177405580418  c
 -11.31346461600294    4.31431629079931   -0.29776165230253  c
  -9.17940750954931   -3.00725443644106    0.30494723399283  f
 -14.09790330352151   -1.73341865777035    1.27945213492892  f
```

```
  -15.44382202512921     3.22330018267177     0.84697759908452  f
  -11.90813815068515     6.71561173327124    -0.52071965594901  f
   -7.08651157469176     5.06647992191678    -1.41574214891018  f
$end
```

E_{tot} = -1915.969171034 H

7.7 FXe(NC$_5$F$_5$) (neutral)

```
$coord
   -5.47171138180923     2.05569228167577     0.16560442860614  xe
   -9.72608754263850     2.05825691647547     1.67199190440498  f
    1.13326130084258     2.42501798917257    -0.69753341857361  n
    2.32604210027625     4.57634879622596    -0.43777935909972  c
    2.40529855352507     0.31169696648235    -0.51604578307894  c
    4.98357216725947     0.21543393889358    -0.05950894820586  c
    6.24430241030345     2.50273318804362     0.21403834082405  c
    4.89954622606115     4.75031026868132     0.02343793225209  c
    0.97649507376237     6.68405995634863    -0.62832824207259  f
    6.06374525578339     6.96147960763773     0.27910952962892  f
    8.70427641427075     2.54034729217911     0.65346307136411  f
    1.13377476285291    -1.83650345577571    -0.78460794451657  f
    6.22824331493255    -1.95939936948456     0.11615848846678  f
$end
```

E_{tot} = -1173.602630286 H

7.8 [FXe(NC$_5$F$_5$)]$^+$

```
$coord
    0.00000000000000     0.00000000000000    -6.03065628971453  xe
    0.00000000000000     0.00000000000000    -9.69793769483740  f
    0.00000000000000     0.00000000000000    -1.57341132016021  n
    2.18640926668082     0.00000000000000    -0.31613785885661  c
   -2.18640926668082     0.00000000000000    -0.31613785885661  c
   -2.28163211036844     0.00000000000000     2.29430407355900  c
    0.00000000000000     0.00000000000000     3.61707597538799  c
    2.28163211036844     0.00000000000000     2.29430407355900  c
    4.26076352700110     0.00000000000000    -1.67537709609209  f
    4.45437155835922     0.00000000000000     3.49283372870989  f
    0.00000000000000     0.00000000000000     6.08035793025653  f
   -4.26076352700110     0.00000000000000    -1.67537709609209  f
   -4.45437155835922     0.00000000000000     3.49283372870989  f
$end
```

E_{tot} = -1173.281110156 H

7.9 N(C$_6$H$_2$Br$_3$)$_3$

```
$coord
    1.11712075149124    -0.94720007433793    -0.00188636357591  n
    1.25962794596811     0.50585617907827     2.23801999800081  c
   -0.41803438788786     0.14041737702127     4.26331467183723  c
    3.08760991369692     2.40212195217114     2.57361924206142  c
   -0.26404294297216     1.52006786298604     6.48160343349493  c
    3.21381826089401     3.86409079077654     4.74032761156588  c
    1.54425637398222     3.39357607121406     6.69384432358017  c
   -1.58768103177591     1.16975565121154     7.99037658716485  h
    4.64475997723536     5.30415817473934     4.91285157230641  h
    1.14449600046671     0.26391158833658    -2.38544461610592  c
```

2.74887895621986	-0.52519557127343	-4.34838736185947	c
-0.43118463287609	2.32924364519688	-2.93450272267355	c
2.75628132553808	0.61814607521589	-6.70223393094377	c
-0.38552743135508	3.55568864950250	-5.24569516727128	c
1.19845210959501	2.67207757943841	-7.12591189272273	c
4.01509197459301	-0.05584970083894	-8.15559687409986	h
-1.62389495876812	5.13843302963316	-5.58106309539891	h
0.94773463089014	-3.61173683495483	0.14190558804650	c
-0.86841543785293	-4.98381829222628	-1.22519818987902	c
2.58524524544765	-5.04769501661572	1.66062986983228	c
-1.01947348985795	-7.59466176237080	-1.13272355611027	c
2.40480208180913	-7.65149238700254	1.84939865134339	c
0.61076398799364	-8.91071158151670	0.42785350618750	c
-2.43982320352766	-8.56975739133702	-2.22034734177355	h
3.69806087914471	-8.67592554548975	3.04487015198031	h
-2.89425671644097	3.52304979395247	-0.60905567442669	br
5.18078324866946	-3.12929424756355	-3.89656036592722	br
1.23485807984660	4.29895754244809	-10.32835639391411	br
5.31124150457299	-3.56087022579198	3.46560157607396	br
-3.39674622058303	-3.36655607661474	-3.19787656442917	br
0.38333438024017	-12.49048224235626	0.62078336715764	br
5.66520255061622	3.01992106974002	0.15001784551988	br
-3.16313666230542	-2.16794717855712	4.07594232920893	br
1.73708704297961	5.34384354771732	9.70432183768527	br

$end

E_{tot} = -23910.58182631 H

7.10 $[N(C_6H_2Br_3)_3]^+$

$coord

1.11744566245537	-0.94768661404032	-0.00160708103368	n
1.25860949845506	0.49209903744489	2.21780410478709	c
-0.38381070512065	0.03633605520653	4.28182704773625	c
3.04944826292962	2.46305088233134	2.48953013083033	c
-0.23533985361899	1.44184153780544	6.46956756227728	c
3.17883172073102	3.88775823484554	4.66603956759000	c
1.54092032086795	3.36910209988034	6.65391289175230	c
-1.52970224809330	1.08208220470808	8.00222377319759	h
4.58196416551875	5.35546408522148	4.84188851032071	h
1.14439084254105	0.25245172653682	-2.36332845880443	c
2.70191351382947	-0.62535158470869	-4.35558129412114	c
-0.38480137978209	2.39342979921439	-2.85647050309454	c
2.71784681393371	0.54597934912388	-6.68183060932114	c
-0.34739427587866	3.58248041360182	-5.17345590694784	c
1.19848846795127	2.65162902398909	-7.08319118041227	c
3.94611928947116	-0.13610336997805	-8.15830174028690	h
-1.55459837020912	5.18874321171418	-5.51463878538311	h
0.94938882294433	-3.58787611312076	0.14068257802899	c
-0.82873695759329	-4.95233578394024	-1.32288558053383	c
2.55054143263885	-5.00183915143027	1.75388938469433	c
-0.98393669809596	-7.54908699443572	-1.19448009042601	c
2.37587426294828	-7.59619328260967	1.90414261630800	c
0.61375244857239	-8.86447694891513	0.42434376608458	c
-2.37650560563025	-8.54321013849682	-2.30187238953138	h
3.63982714943626	-8.63436117138683	3.12007066130922	h
-2.80021567103439	3.63195261988536	-0.52414171078126	br
5.08585002410317	-3.26174273737224	-3.93813725405565	br
1.23485878514028	4.25990487013323	-10.24682389326588	br

```
  5.23260037533083    -3.52499360284058     3.60682749294371  br
 -3.31591979973693    -3.35477418782939    -3.34020703411123  br
  0.38857796296878   -12.40135515914591     0.61472196967484  br
  5.58558488859506     3.13043599673825     0.05427376593603  br
 -3.08488756897746    -2.30661223825148     4.13213629823570  br
  1.73030452809622     5.29738038165328     9.62751344233935  br
$end
```

E_{tot} = -23910.31268384 H

7.11 C_5F_5N

```
$coord
 -12.19180168410231     2.75647945000392     0.00000539344407  c
  -9.71107102360147     3.61521373360031    -0.00000283523646  c
  -7.81257201967956     1.80798862503720     0.00000621802465  c
 -12.66247133377423     0.17388401848336    -0.00000081064892  c
 -10.58708161183762    -1.42704111440502     0.00000593622432  c
  -8.24887777728573    -0.62510430706624     0.00002536628694  n
 -10.97188971675136    -3.90845919986912    -0.00001005774722  f
 -15.02228297722134    -0.69470554334963    -0.00002133980135  f
 -14.09092460233462     4.38505197073991     0.00002266174139  f
  -9.21223859258305     6.07982508590600    -0.00001888295968  f
  -5.41872480350805     2.56633762369642    -0.00001164932777  f
$end
```

E_{tot} = -744.4778984039 H

7.12 $C_5H_3F_2N$

```
$coord
 -24.32281413440763     4.18641981741228     0.00580451554585  c
 -26.61982845198082     2.91707597622866     0.00193912905469  c
 -26.50575215205987     0.30110861713185     0.00491736690363  c
 -24.42256206268934    -1.04292839212487     0.01082411770492  n
 -22.28963364525752     0.22070658083004     0.01388433978772  c
 -22.07586975152141     2.83042648740724     0.01165503271869  c
 -24.28385600087877     6.22949548724486     0.00447310159882  h
 -28.41608153711007     3.88144051546014    -0.00355400667913  h
 -28.66348618536754    -1.01505177275607     0.00179003735346  f
 -20.18369748738849    -1.17680798491878     0.01975916684165  f
 -20.24415617648349     3.72563532301064     0.01486768344261  h
$end
```

E_{tot} = -446.7594963453 H

7.13 $[Xe(OTeF_5)(NC_5H_3F_2)]^+$

```
$coord
  -8.04140578056800     0.26008780589980     0.51755457628852  xe
 -12.53432489372036     0.05225615824782     0.26047050498456  n
  -4.20936078268595     0.19971605629345     0.84300372989350  o
  -2.15604450681872     3.03747720285909    -0.21634817084694  te
   0.02455087756871     0.80255838723455    -1.75516530701342  f
  -0.37883513890972     2.78524712492928     2.76664606295521  f
  -4.44045637122533     5.24958394839487     1.29633164080732  f
  -4.04419771391447     3.29044195648440    -3.17769190924266  f
  -0.21138019508291     5.72670186450511    -1.22383985325849  f
 -13.71497065252177    -2.17090488579439     0.08383401882565  c
 -13.91765090458469     2.16281218876706     0.28373795842848  c
```

-16.51390742634978	2.16043735136333	0.13434306690376	c
-17.70308170862765	-0.17557396357964	-0.04839917824475	c
-16.30040420832839	-2.39757453016380	-0.07612058316975	c
-12.61203020875461	4.27869031016143	0.46186823256526	f
-19.74044006101372	-0.26543049830192	-0.17018846756356	h
-17.15035859362420	-4.24681654270633	-0.21532812193821	h
-12.21631856702277	-4.16364272049543	0.07324939722497	f
-17.53321861014056	3.92725010907894	0.16202350513928	h

$end

E_{tot} = -1618.269519171 H

8. References

1. K. F. Hoffmann, A. Wiesner, S. Steinhauer and S. Riedel, *Chem. Eur. J.*, 2022, DOI: 10.1002/chem.202201958.
2. W. Tötsch and N. Bartlett, *J. Fluorine Chem.*, 1982, **21**, 32.
3. F. Sladky, *Angew. Chem. Int. Ed. Engl.*, 1969, **8**, 523.
4. F. Sladky and C. J. Schack in Inorganic Syntheses, ed. J. M. Shreeve, John Wiley & Sons, New York, Chichester, Brisbane, Toronto and Singapore, 1st edn, 1986, vol. 24, ch. 1, pp. 33–37.
5. R. K. Harris, E. D. Becker, S. M. Cabral de Menezes, P. Granger, R. E. Hoffman and K. W. Zilm, *Magn. Reson. Chem.*, 2008, **46**, 582.
6. P. H. M. Budzelaar, gNMR (5.0.6.0) NMR Simulation Program, IvorySoft, 2006.
7. P. Pröhm, W. R. Berg, S. M. Rupf, P. Voßnacker, S. Riedel, *Chem. Eur. J.*, 2021, **27**, 17676.
8. G. M. Sheldrick, *Acta Cryst. A*, 2008, **64**, 112.
9. G. M. Sheldrick, *Acta Cryst. C*, 2015, **71**, 3.
10. O. V. Dolomanov, L. J. Bourhis, R. J. Gildea, J. A. K. Howard and H. Puschmann, *J. Appl. Cryst.*, 2009, **42**, 339.
11. TURBOMOLE V7.6.1 2022, a development of University of Karlsruhe and Forschungszentrum Karlsruhe GmbH 1989-2007, TURBOMOLE GmbH since 2007.
12. (a) A. D. Becke, *J. Chem. Phys.*, 1993, **98**, 5648. (b) C. Lee, W. Yang and R. G. Parr, *Phys. Rev. B*, 1988, **37**, 785. (c) S. H. Vosko, L. Wilk and M. Nusair, *Can. J. Phys.*, 1980, **58**, 1200. (d) P. J. Stephens, F. J. Devlin, C. F. Chabalowski and M. J. Frisch, *J. Phys. Chem.*, 1994, **98**, 11623.
13. M. Sierka, A. Hogekamp and R. Ahlrichs, *J. Chem. Phys.*, 2003, **118**, 9136.
14. F. Weigend and R. Ahlrichs, *Phys. Chem. Chem. Phys.*, 2005, **7**, 3297.
15. S. Kotsyuda, A. N. Toraman, P. Voßnacker, M. A. Ellwanger, S. Steinhauer, C. Müller and S. Riedel, *Chem. Eur. J.*, 2023, DOI: 10.1002/chem.202202749.
16. H. P. A. Mercier, M. D. Moran, J. C. P. Sanders, G. J. Schrobilgen and R. J. Suontamo, *Inorg. Chem*., 2005, **44**, 49.
17. K. P. Huber, G. Herzberg, Molecular Spectra and Molecular Structure. IV. Constants of Diatomic Molecules, Van Nostrand Reinhold Co., 1979
18. S. G. Lias, J. E. Bartmess, J. F. Liebman, J. L. Holmes, R. D. Levin, W. G. Mallard, *J. Phys. Chem. Ref. Data*, *Suppl. 1*, 1988, **17**, 1.
19. W. Schmidt, E. Steckhan, Chem. Ber., 1980, **113**, 577.
